# Supplementary figures and images for: Bacterial dormancy: A subpopulation of viable but non-culturable cells demonstrates better fitness for revival
Source: PLoS Pathog. 2021 Jan 13;17(1):e1009194. doi: 10.1371/journal.ppat.1009194 (PMC7837498; doi:10.1371/journal.ppat.1009194)

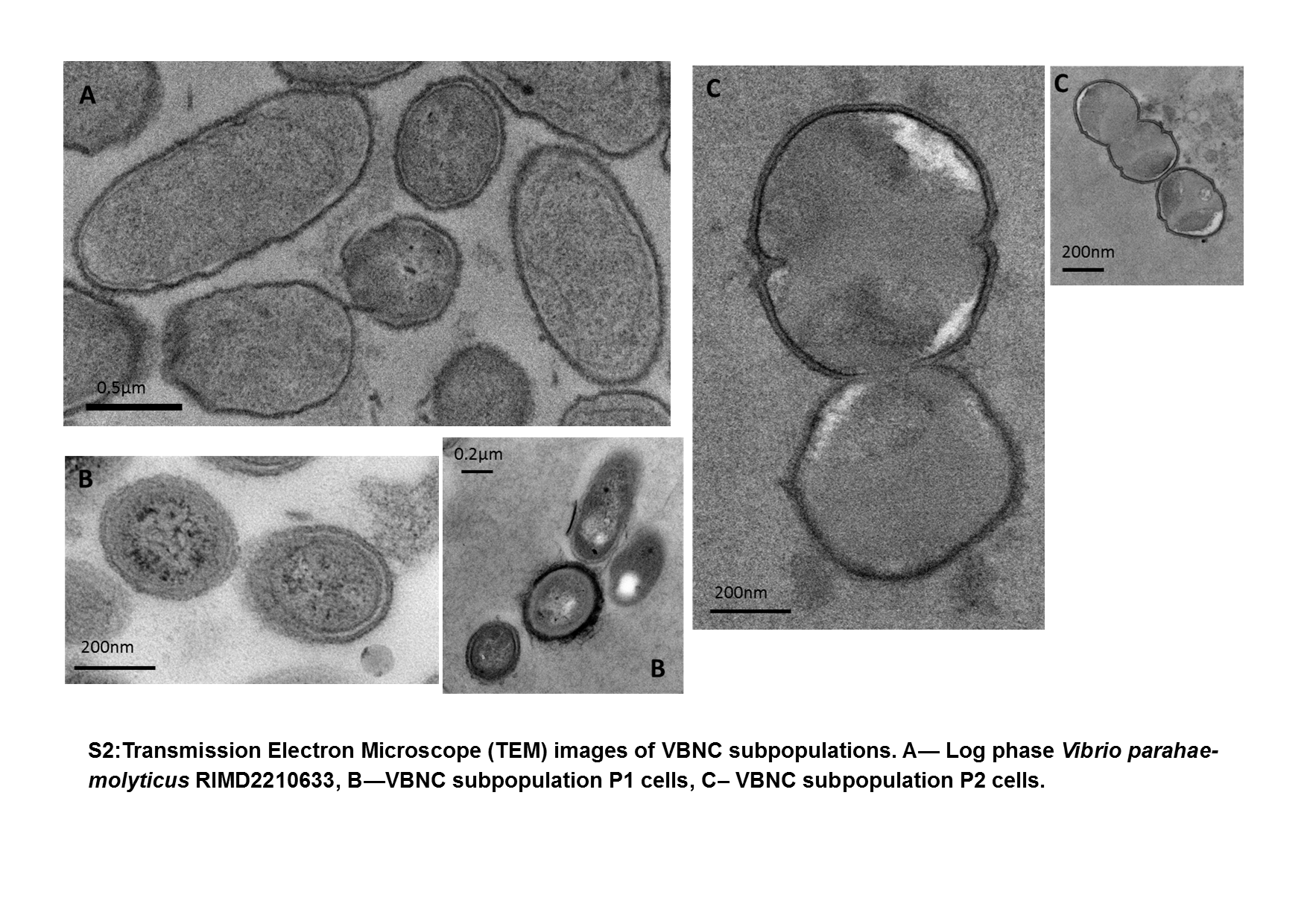

Supplement: S1 Fig — (TIF) [file ppat.1009194.s002.tif]

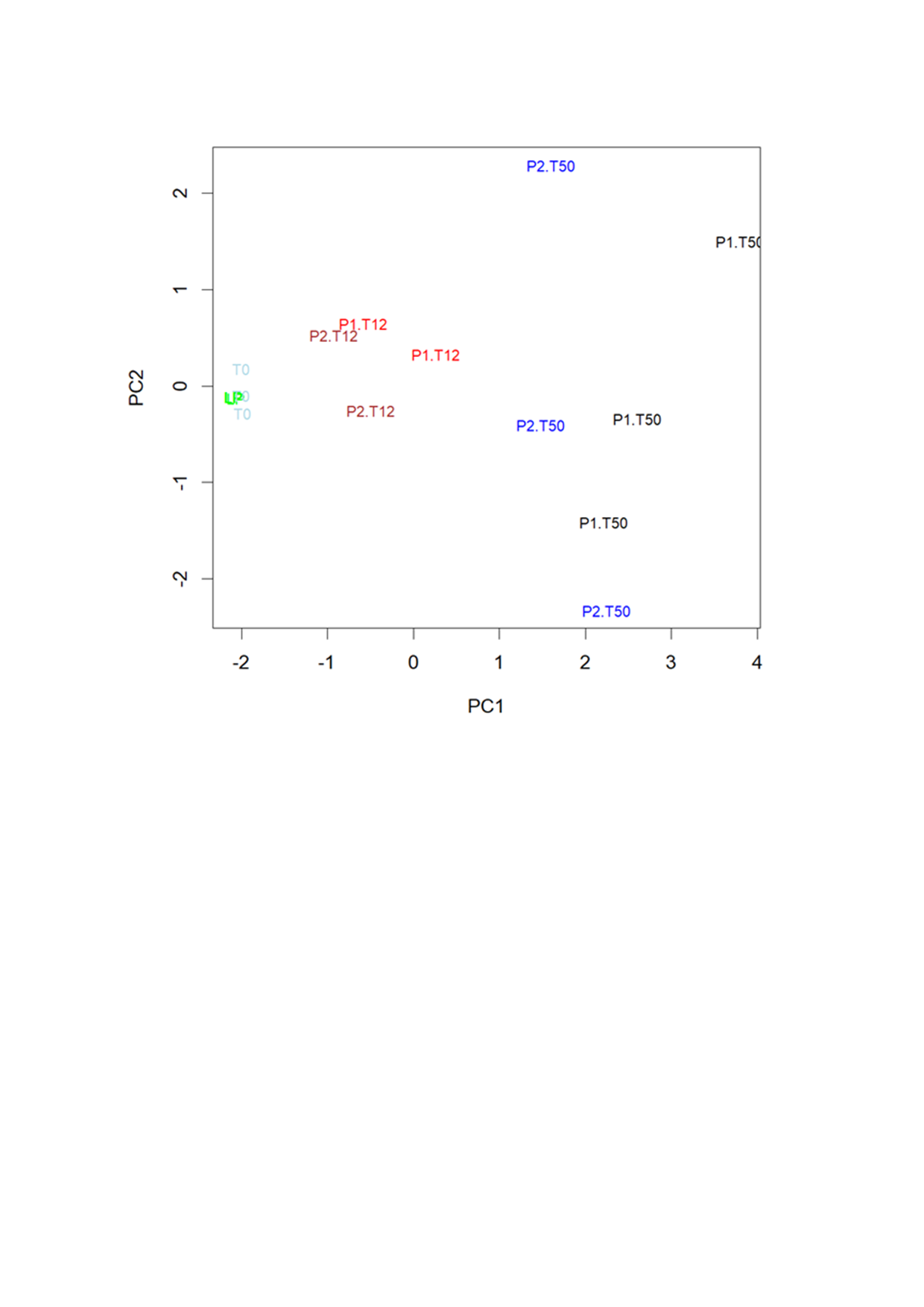

Supplement: S2 Fig — Replicates of the Log Phase cells, T0 and T12 samples are clustered together indicating experimental reproducibility. Replicates of the T50 samples are more distant from each other. (TIF) [file ppat.1009194.s003.tif]

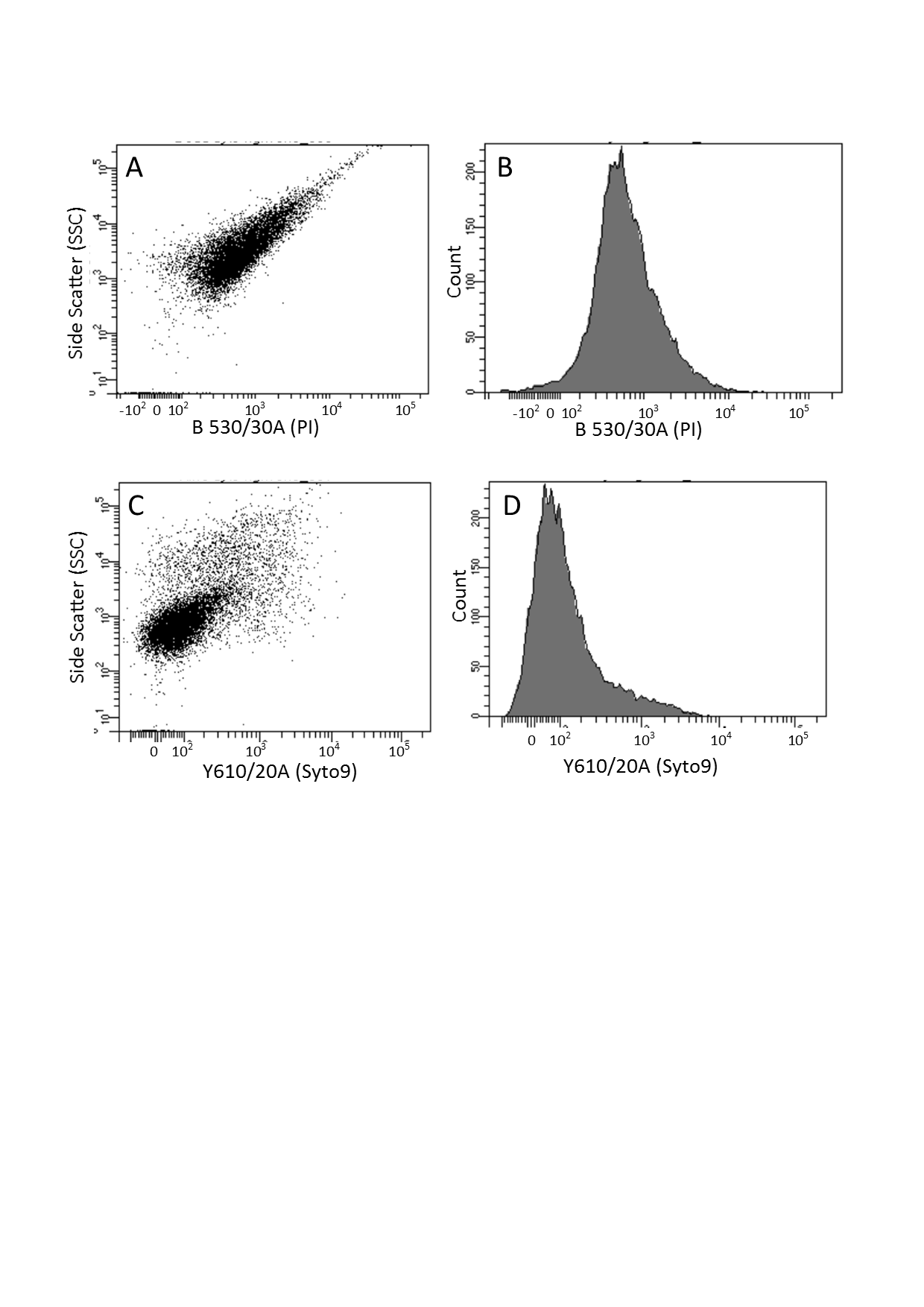

Supplement: S4 Fig — Dot plots (Left) and corresponding histograms (Right) of control experiments using boiled bacterial suspensions (A and B) were used to identify V. parahaemolyticus cells around dead/damaged that had a comprimsed membrane (using propidium iodide (PI) stain). Dot plots (Left) and corresponding histograms (Right) of control experiments using log phase bacteria (C and D) were used to identify V. parahaemolyticus cells that were alive and had an intact cell membrane (using Syto9 stain). (TIF) [file ppat.1009194.s005.tif]

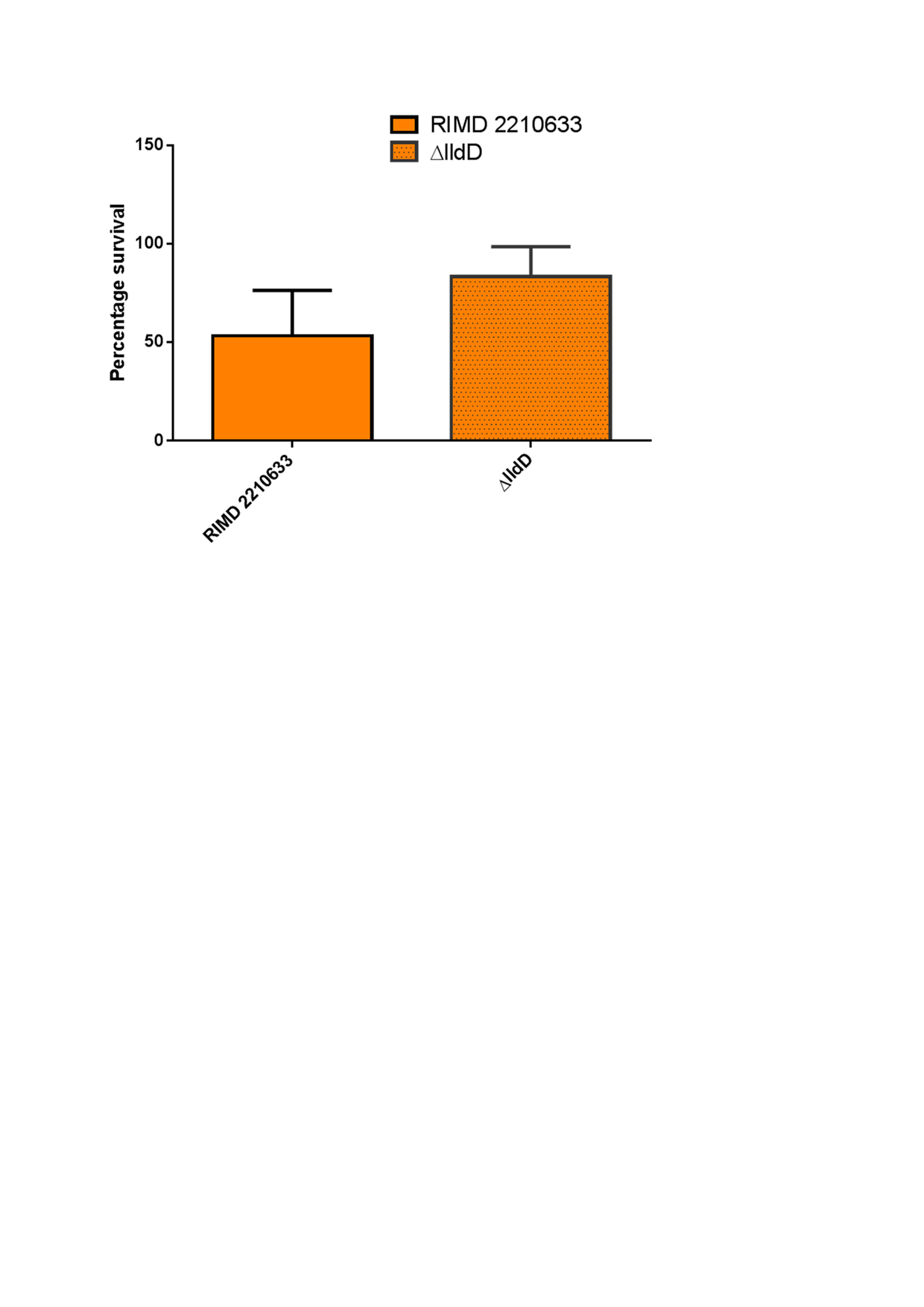

Supplement: S5 Fig — A dose of 105 CFU of RIMD2210633 or RIMD2210633:ΔlldD CFU was injected into larvae. Percentage survival was measured after 48 hours. There was no significant difference between virulence of the wildtype and the RIMD2210633:Δlld. (TIF) [file ppat.1009194.s006.tif]

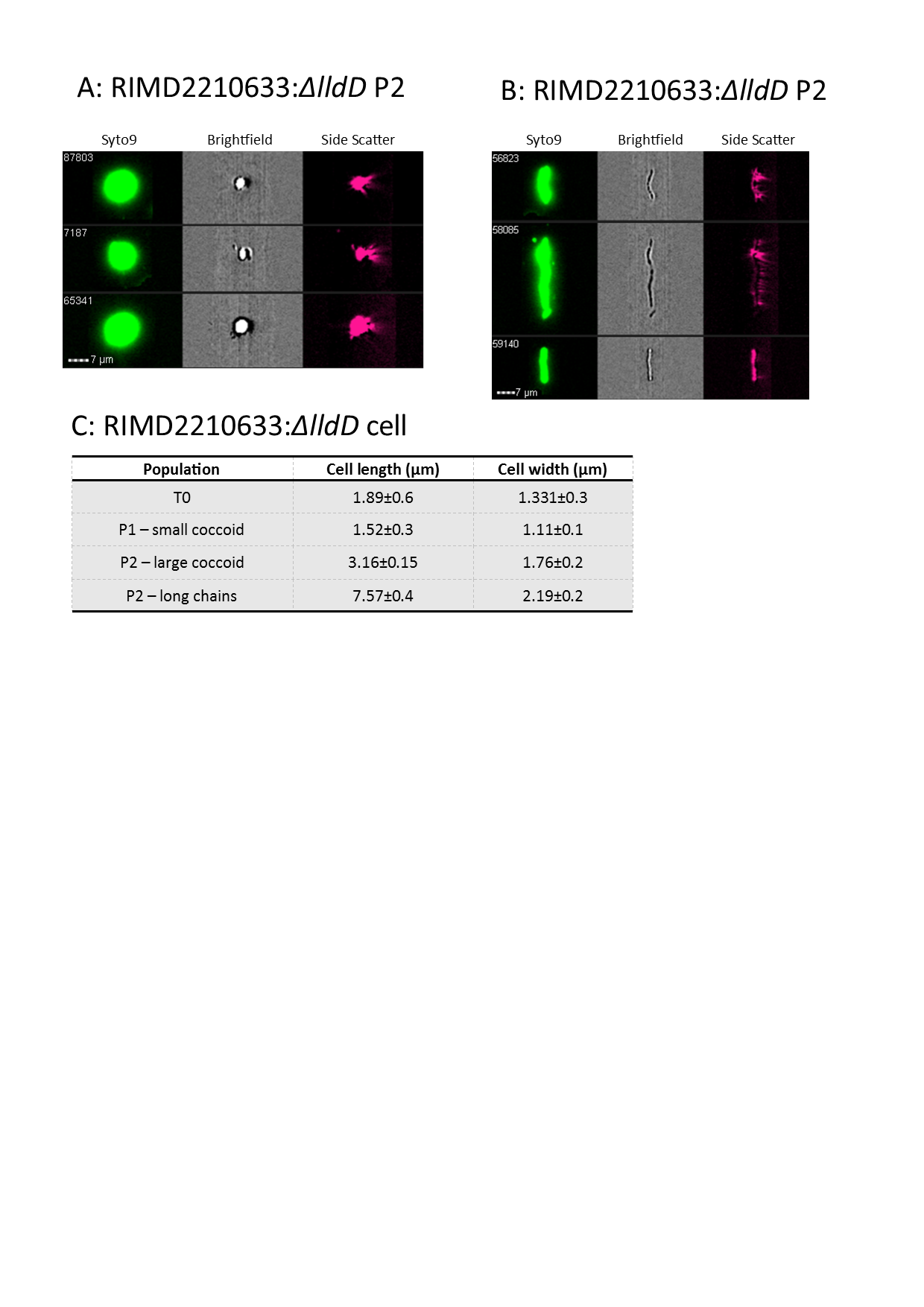

Supplement: S6 Fig — Microcosms of the mutant RIMD2210633:ΔlldD were prepared and allowed to enter VBNC state. After 12 days in the VBNC cells were stained with Syto9 and examined for morphology using Imagestream Technology. Panel A and B show cells of the P2 population that were large coccoid or long filaments respectively. Panel C is a table indicating the cell lengths and widths of the cells. (TIF) [file ppat.1009194.s007.tif]
