# Supplementary material for: Bacterial dormancy: A subpopulation of viable but non-culturable cells demonstrates better fitness for revival
Source: PLoS Pathog. 2021 Jan 13;17(1):e1009194. doi: 10.1371/journal.ppat.1009194 (PMC7837498; doi:10.1371/journal.ppat.1009194)
Supplement: S1 Table — (DOCX) [file ppat.1009194.s008.docx]

**S3: Protein data and the numbers of proteins detected in each group.**

| Sample | Number of proteins detected |
| --- | --- |
| T0 | 1533 |
| P1-T12 | 1477 |
| P2-T12 | 1497 |
| P1-T50 | 1444 |
| P2-T50 | 1449 |
