# Supplementary material for: Bacterial dormancy: A subpopulation of viable but non-culturable cells demonstrates better fitness for revival
Source: PLoS Pathog. 2021 Jan 13;17(1):e1009194. doi: 10.1371/journal.ppat.1009194 (PMC7837498; doi:10.1371/journal.ppat.1009194)
Supplement: S2 Table — Determined by regression analysis. Mean of the normalised abundance values were used with each group. (DOCX) [file ppat.1009194.s009.docx]

**S5. Correlation between the proteomes of the analysed groups.** Determined by regression analysis. Mean of the normalised abundance values were used with each group.

| **Sample comparisons** | **Adjusted R^2^ of linear regression** | **Slope of the linear regression line** |
| --- | --- | --- |
| **T0 vs P2-T12** | 0.601 | 0.718 |
| **T0 vs P1-T12** | 0.494 | 0.596 |
| **T0 vs P2-T50** | 0.351 | 0.397 |
| **T0 vs P1-T50** | 0.258 | 0.327 |
| **P2-T12 vs P1-T12** | 0.796 | 0.817 |
| **P2-T50 vs P1-T50** | 0.799 | 0.859 |
| **P2-T12 vs P2-T50** | 0.524 | 0.524 |
| **P1-T12 vs P1-T50** | 0.555 | 0.565 |
