# Supplementary material for: Bacterial dormancy: A subpopulation of viable but non-culturable cells demonstrates better fitness for revival
Source: PLoS Pathog. 2021 Jan 13;17(1):e1009194. doi: 10.1371/journal.ppat.1009194 (PMC7837498; doi:10.1371/journal.ppat.1009194)
Supplement: S3 Fig — Protein expression data is shown only if the fold change was significantly different in the VBNC populations, compared to protein expression in the P0 population. Heat map shows log2 fold change difference in protein expression: highly downregulated–dark blue, highly upregulated–yellow. -9.9 or 9.9 log2 fold change values were assigned empirically to the protein if the protein was not detected in the VBNC population or in the T0 population, respectively. Locus tags of proteins which were significantly upregulated or significantly downregulated in all VBNC populations are highlighted with green or red, respectively. (PDF) [file ppat.1009194.s004.pdf]

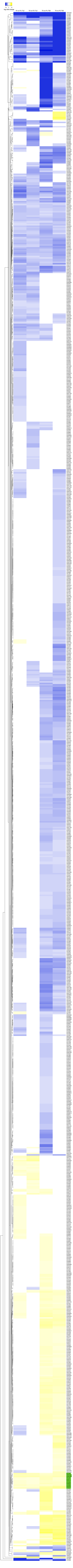

−10
0
10
log2 fold change
T0 vs P1-T12
T0 vs P2-T12
T0 vs P1-T50
T0 vs P2-T50
