# Supplementary material for: Bacterial dormancy: A subpopulation of viable but non-culturable cells demonstrates better fitness for revival
Source: PLoS Pathog. 2021 Jan 13;17(1):e1009194. doi: 10.1371/journal.ppat.1009194 (PMC7837498; doi:10.1371/journal.ppat.1009194)
Supplement: Attachment — Submitted filename: Reviewers responses November 2020 v2.docx [file ppat.1009194.s012.docx]

|  | Biosciences,  College of Life & Environmental Sciences,  Geoffrey Pope Building,  Stocker Road,  Exeter, UK.  EX4 4QD  t +44 (0)1392 725177  e s.wagley@exeter.ac.uk  w biosciences.exeter.ac.uk |
| --- | --- |

Associate Editor,

PLOS Pathogens,

19^th^ November 2020

Dear Professor Baumler,

**Ref: PPATHOGENS-D-20-01971**

**Title: Bacterial dormancy: a subpopulation of viable but non-culturable cells demonstrates better fitness for revival.**

Thank you for forwarding the valuable comments from the reviewers. We are impressed by the number and quality of the reviews, and clearly this indicates the level of interest in this work. We have now considered all of these comments and carried out additional work where necessary. Our responses to the reviewers’ comments are detailed, on a point-by-point basis.

One comment (point 2) from reviewer #2 suggested we look at other test conditions that would induce the VBNC state and check whether similar subpopulations of VBNC cells would be created under such different induction conditions. In this study, we used low nutrient and temperature as a way of inducing the VBNC state. We agree that it would be good to look at other induction methods such as UV damage, pH or another type of stress but we feel that such experiments may take months to set up and get the conditions correct and so we do not think it would be feasible in the scope of this study.

A comment from reviewer #3, which we were also unable to address, was that we did not look at different *V. parahaemolyticus* strains and only focussed on the reference strain RIMD2210633. We chose the reference strain RIMD2210633 because it is well studied, it is typical of O3:K6 pandemic clinical strains in that it is TDH positive and TRH negative, it has been fully genome sequenced and has served as a prototypic strain in a number of studies. Thus, we believe using clinical strain RIMD2210633 is a relevant strain to studying the virulence potential of *V. parahaemolyticus*. Although it would be good to analyse the virulence potential of VBNC states in other strains of *V. parahaemolyticus* we do not feel that we would have enough time or resources to do this adequately in the scope of this study.

Several of the minor comments were raised around the further characterisation of the Δ*lldD* mutant, which was not possible in March this year because of the enforced shutdown of the University labs in the UK. However, we did anticipate questions from reviewers around this and started experiments as soon as labs were open again and so we have addressed as many of the comments around this issue that we can.

I hope that you, the other Editors and reviewers agree that our manuscript not only reports the first finding that populations of dormant cells are made up of subpopulations, but also that they differ in both their ability to be resuscitated and their proteome profile. We believe these findings will make a major contribution to the field of bacterial dormancy and it is important that they are published in a leading journal such as PLOS Pathogens.

Details regarding the changes we have made to the manuscript to address the reviewers concerns are listed below. We hope that the manuscript will now be accepted for publication.

Yours sincerely,

Dr Sariqa Wagley.

**Major comments**

Reviewer 1: We thank reviewer #1 for their comments and have made the following changes.

1. For the flow cytometry please present the live and dead (boiled) controls. This would help the reader understand what was done, and how the gates were set. In addition, the result section should be revised. My understanding is that PI was used to test for viability, which was detected by Y610/20. P2 is therefore less viable than P1, but the author do not consider P2 as dead. Again, the controls (live and dead) would help the reader interpret this important dataset. Why not used Syto 9 signal instead of SSC?

**Boiled and log phase cultures were used to set the gates and this information has now been included in the supplementary section as S9 Figure and referenced to in Lines 608-610. We agree with the reviewer that if PI was used to set viability then P2 would be less viable. However, we used the Syto9 signal to determine viability and this signal was detected at Y610/20 while the PI signal was detected at B530/30. We have changed Figure 2, so that the X axis now reads Y610/20A (Syto9) in order to reduce confusion. We also realise that confusion may have arisen by our description in the methods section at lines 604-606 that read “Particle fluorescence was measured at 488 nm excitation, 530 ± 30 nm emission for the RSG and Syto9 stain, and 561 nm excitation, 610 ± 20 nm emission for the PI stain”. We have now changed this to read “Particle fluorescence was measured at 488 nm excitation, 530 ± 30 nm emission for the PI stain, 610 ± 20 nm emission for the Syto9 stain.”. We apologise for this error in the methods section and any confusion it has caused in interpreting the results.**

**During all FACS analysis, we had several plots open for analysis, which included Side Scatter (SSC) vs Forward Scatter (FSC), SSC vs PI, and SSC vs Syto9. We saw greater discrimination based on SSC and therefore used SSC vs Syto9 (Y610/20) to collect the P2 cells from the population.**

2. Was the flow cytometry done on 14 days stored culture or 5 day old culture (in reference to Fig. 1).

**All data presented after Figure 1 was carried using cultures that were ≤5 days old. To clarify this we highlight the Line 150: “For subsequent experiments we used VBNC microcosms established from cultures that were less than 5 days old (Fig 1A)”**

3. I am not convinced that the seafood samples contained Vv. I presume that the shrimps harbour a variety of microorganisms that could results in similar images. How does the author confirm that the cells the see are Vv? Microscopic morphology cannot be used to identify microorganisms, unless some kind of specific labelling was used (such as antibodies but this does not seem to be the case).

**The shrimp samples were processed using aseptic techniques and not found to contain *V. parahaemolyticus* unless it was pre-warmed. We confirmed their identity by firstly subculturing on to selective media TCBS agar and secondly by screening a number of colonies by PCR for species marker toxR. We tested this sample 3 times in this way with the same results and this demonstrates that *V. parahaemolyticus* were in the VBNC state. The *V. parahaemolyticus* strain identified from the shrimp sample was TDH and TRH negative when tested by PCR. However, we do agree that we do not know that cells we see using imaging flow cytometry are *V. parahaemolyticus*.**

**We have now deleted lines 282-284 and added in lines 291-296 to now read “Using IFC images alone we cannot be certain that the cells we see are *Vibrio* species however, they do resemble *V. parahaemolyticus* VBNC cells. Cells from P1 and P2 gated areas were collected using FACS and non-resuscitated cells could not be grown on agar while those treated with the resuscitation step were culturable and were identified as *V. parahaemolyticus* (Fig 5F) using ToxR species targeted PCR.” to address the reviewers comment.**

4. In general, I find that the figures are of poor quality (grainy, font too small) and the legends could be significantly improved. I cannot read protein label on the proteome figures. The authors needs to describe what the experiment is and provide sufficient details for the reader to understand the figure without having to constantly refer to different part of the text.

**We agree with the author here and have now provided figures images with higher resolution and larger font size.**

**For Figure 6 : We have increased the size of the pictures so that the categories of proteins are readable.**

**For Figure 7, we have increased the font size of the 11 proteins that were significantly upregulated in VBNC populations. To make the image less grainy we have changed the colour of the downregulated proteins as grey in colour from the original black. We have also included in the figure legend details for the reader about the experiment and have added in the line “Using quantitative mass spectrophotometry, the proteome of VBNC populations P1 and P2, derived from time points T12 and T50, was resolved and compared to that from cells at T0 time point.”**

5. Several section of the results needs to be rewritten. The text is disjointed, the panels are not presented in the same order in the text and in the Figure and some panels are not presented at all (Fig 4B) and (Fig. 8B). It was very difficult for me to understand what was done. I suggest that the author present the experiments, and the important details, before presenting the results of it. In particular, the microcosm should be presented (conditions, medium, etc) as well as the general method to test resuscitation (PBS). I find the use of T12 and T50 confusing (as it seems to relate to time after the population is composed of VBNC cells only) rather than days. Also Fig 1B present data as VBNC+number of days. I presume VBNC is a specific time point but this is unclear.

**We apologise for these errors and have rectified them in the revised text. We have made the following changes.**

**We have changed Line 167 and inserted the words “and Fig 2B” to make reference to this dataset.**

**We have changed Line 173 from ‘’Fig 2B and 2C” to read “Figure 2C and 2D”.**

**In order to address the issue of presenting data in the order that it appears we have changed the following lines 197-201 that previously read “The P2 subpopulation contained large coccoid cells that were 6.34 ± 1.1 µm in length and 4.3 ± 0.48 µm in width and accounted for 10% of the microcosm population (Fig 3D). Subpopulation P2 also contained large filaments or chains of rods that accounted for 0.5% of the microcosm and had an average length 2.69 ± 0.6 µm and an average width of 1.51 ± 0.5 µm (Fig 3C).” to now read “The subpopulation P2 contained large filaments or chains of rods that accounted for 0.5% of the microcosm and had an average length 2.69 ± 0.6 µm and an average width of 1.51 ± 0.5 µm (Fig 3C). The subpopulation P2 also contained large coccoid cells that were 6.34 ± 1.1 µm in length and 4.3 ± 0.48 µm in width and accounted for 10% of the microcosm population (Fig 3D).”.**

**We have also changed the order of the panels in Figure 4 to reflect the order in which they appear in the text in the manuscript. We have changed the legend of Figure 4 on lines 255-258 from “Panel A shows of *Galleria mellonella* after 48 h when injected with different VBNC cell types. Panel B shows a heat map identifying regulation among known virulence proteins. Panel C shows SEM pictures of VBNC cells with extracellular matrix attached surrounding the cells.” to now read “Fig 4: Virulence potential of *V. parahaemolyticus* RIMD2210633 cell types. Panel A shows SEM pictures of VBNC cells with extracellular matrix attached surrounding the cells. Panel B shows survival of *Galleria mellonella* after 48 h when injected with different VBNC cell types. Panel C shows a heat map identifying regulation among known virulence proteins”. For continuity, we have also changed line 217 from “Fig 4C” to read “Fig 4A” and Line 248 and Line 254 from “Fig 4” to read “Fig 4B” and line 265 from “Fig 4C” to read “Fig 4A” and line 273 from “Fig 4” to read “Fig 4B” and Line 504 from “Fig 4B” to now read “Fig 4A”.**

**Finally, we have checked the text relating to Figure 8 and noticed an error in the reference. We have now changed Line 399 from “Fig 8D” to read “Fig 8B”. We have also moved the line “LldD is responsible for converting L-lactate to pyruvate (Fig 8D and 8E)” to the end of the paragraph so that the numbers follow through**.

6. What is Fig. 4B?

**We apologise as we realise after reading the reviewers comments that we omitted, within the results section, an explanation for heat map Figure 4C (previously Figure 4B). To resolve this we have inserted text at line 275-276 “Fig 4C shows a heat map of regulation among virulence proteins and indicates no major known virulence mechanisms where upregulated”.**

Reviewer 2: We thank reviewer #2 for their comments and have made the following changes to address their concerns.

1. Can the author provide possible explanation to why P2 has high PI signal but intact membrane?

**The subpopulation P2 does not have a higher PI signal; it has a high Syto9 signal. We apologise for this confusion and have changed Figure 2 so that the X axis now reads Y610/20A (Syto9). We realise also that this confusion may have arisen by our erroneous description in the methods section at lines 604-606 that read “Particle fluorescence was measured at 488 nm excitation, 530 ± 30 nm emission for the RSG and Syto9 stain, and 561 nm excitation, 610 ± 20 nm emission for the PI stain”. We have now corrected this to read “Particle fluorescence was measured at 488 nm excitation, 530 ± 30 nm emission for the PI stain, 610 ± 20 nm emission for the Syto9 stain.”.**

2. It is unclear whether the presence of distinct VNBC populations is specific to the condition tested in the manuscript. The authors should test whether distinct VNBC populations are also present when VNBC state is induced by a different stimulus, in order to support their claim (line 484-486).

**We agree with the reviewer that the presence of the distinct VBNC populations seen in this study may be a consequence of the conditions used to induce the VBNC state (that were low nutrient and low temperature). We agree that testing other induction methods would be valuable in understanding if VBNC subpopulations exists universally. However, this is beyond the scope of the current study. We did however confirm the presence of these subpopulations in a ‘real world’ model after testing a seafood sample known to harbour VBNC cells and here we similarly saw VBNC subpopulations exists. We have now changed the lines 515-517 from “To the best of our knowledge, we report unequivocally that different populations of VBNC cells exist and this is the first report of the quantification of these different VBNC cells and their different resuscitation potentials.” To now read “To the best of our knowledge, we report unequivocally that different populations of VBNC cells exist** **(under the VBNC induction conditions tested in this study) and this is the first report of the quantification of these different VBNC cells and their different resuscitation potentials.”**

3. The authors suggested that VBNC subpopulations cannot be resuscitated inside *G. mellonella*, and that the larvae death was caused by the virulent compounds associated with the whole microcosm. A direct way to test this hypothesis will be to infect the larvae with FACS-sorted P1 or P2 population without resuscitation.

**We agree with the reviewer and indeed show this in Figure 4B. Here, we have shown that VBNC cells from T12 from both P1 and P2 were washed and injected into larvae. This is labelled in the graph as pre-resuscitation and the graph shows that pre-resuscitated cells from both P1 and P2 populations did not kill the larvae. To make this clear we have relabelled the graph so that “pre-resuscitation” and “post resuscitation” now reads “without resuscitation” and “with resuscitation”. We hope this finding in the results section is clearer now.**

4. While it is definitely interesting that the addition of pyruvate can resuscitate VBNC, the underlying mechanisms remains elusive. It will be interesting to test the proposed antioxidant mechanism by assaying whether other antioxidant compounds can rescue the ldhA mutant.

**We thank the reviewer for this suggestion, we did test the wild type RIMD2210633 microcosm with other substrates including sodium acetate, sodium pyruvate and sodium lactate. We found that only sodium lactate was able to resuscitate the VBNC cells. We agree with the reviewer that it would be interesting to test the proposed assays on the lldD mutant. We have now tested the lldD mutant with sodium pyruvate, sodium acetate and sodium lactate to see if these compounds are able to resuscitate VBNC cells. We found that none of these compounds including sodium lactate was able to resuscitate the lldD mutant when they were in the VBNC state. To reflect these experiments that were carried out we have changed lines 421-432 from “We investigated whether the addition of sodium lactate would enable the resuscitation of mid-to late stage VBNC cells, when thermal shift methods alone were not sufficient. The addition of 2 mM of sodium lactate resuscitated mid VBNC stage cells (36 days after cells had become unculturable in the microcosm) enabling some cells to be cultured, whilst control VBNC cells incubated in PBS were not resuscitated (Fig 8C).”to now read “We investigated whether the addition of sodium lactate, sodium pyruvate and sodium acetate would enable the resuscitation of mid-to late stage VBNC cells, when thermal shift methods alone were not sufficient. The addition of 2 mM of sodium lactate resuscitated mid VBNC stage cells (36 days after cells had become unculturable in the microcosm) enabling some cells to be cultured, whilst control VBNC cells incubated in PBS were not resuscitated (Fig 8C). The addition of other oxidative compounds including sodium pyruvate and sodium acetate did not resuscitate RIMD2210633 VBNC cells.**

**When we investigated whether the addition of sodium lactate, sodium pyruvate and sodium acetate would resuscitate RIMD2210633:ΔlldD VBNC cells in mid-late stage we found that none of these compounds were able to turn the VBNC cells to culturable forms as seen with the wildtype.”**

Reviewer 3: We thank the reviewer for their comments and have made the following changes to address their concerns.

1. In Figure 4, the authors state that they infected larvae with approximately 10^7 CFU of T0 cells and approximately 10^5 VBNC cells. Later they state that they infected with approximately 10^4 CFU of P1 or P2 sorted cells for infection. It’s difficult to make any conclusions on VBNC virulence given that the authors are using 2-3 logs less VBNC bacteria for infection. The authors should repeat the experiments by normalizing the VBNC cells up to 10^7 or reduce the inoculum of T0 to match the dose being used for VBNC/P1/P2 cells in this Figure.

**We agree with the reviewer that the T0 inoculum should be normalised to match the CFU counts of P1 and P2 cells used. We now show data for larvae survival when 10^5^ CFU/larvae of T0 cells were inoculated (Figure 4). At this dose approximately 50% of the larvae were killed. In comparison with P1/P2 cells that have not been resuscitated there is now a significant difference (P<0.05) indicating that cells in the VBNC state are not virulent.**

**We have now changed lines 247 – 248 from “When we dosed larvae with approximately 10^7^ CFU of T0 cells, all larvae succumbed to infection (Fig 4B)” to “When we dosed larvae with approximately 10^5^ CFU of T0 cells 50% of the larvae succumbed to infection.”**

2. In Figure 4, the authors state that, “V. parahaemolyticus VBNC cells in subpopulation P2, when resuscitated, revert back to virulence capabilities of the log phase bacteria.” However, the authors do not demonstrate directly that virulence is responsible for this observation and it’s not clear whether increased virulence or if growth alone is responsible for reduced larval survival. The authors should address this by repeating the post-resuscitation P2 infection experiment using a non-virulent bacterial mutant and showing that larvae survival is rescued.

**Resuscitation of VBNC cells was carried out in PBS. From previous work we have observed that *V. parahaemolyticus* cannot grow in PBS (data not shown). We have highlighted this in lines 461-463 “As *V. parahaemolyticus* is unable to grow in PBS, we used this medium and temperature upshift to measure the number of cells in the population that could be resuscitated from the VBNC state”. We have now changed lines 486-488 from “In this study, we found conclusive evidence that *V. parahaemolyticus* VBNC subpopulations cannot be resuscitated inside *G. mellonella*. “ to read “In this study, we found conclusive evidence that *V. parahaemolyticus* RIMD2210633 VBNC subpopulations cannot be resuscitated inside *G. mellonella*.”.**

**Minor issues**

Reviewer 1:

1. Line 27: "displayed different abilities", please be more explicit. Which one is better?

**We have now changed line 27 from “The two subpopulations displayed different abilities for revival under favourable conditions.” to now read “VBNC subpopulation P2, had a better fitness for survival under stressful conditions and showed 100% revival under favourable conditions.”**

2. Author summary should be revised. It reads like it was written at the last minute, without revision.

**We have now changed the author summary from “Members of the Proteobacteria are reported to adopt a survival strategy and enter a VBNC state, when exposed to stressful or non-permissible growth conditions. This is a fundamental characteristic employed widely in the natural environment in order for bacteria to survive harsh environmental conditions over a long period to survive. In spite of the importance of the VBNC state in microbiology, we know little about the molecular makeup. In this study, we resolved that distinct subpopulations of bacteria exist in the VBNC state that have a different resuscitation potential and have a distinct proteomic profile. The ability for bacteria to survive in the VBNC state is compounded by their ability to overcome oxidative stress.” to now read “Members of the Proteobacteria are reported to adopt a survival strategy and enter a viable but non culturable (VBNC) state, when exposed to stressful or non-permissible growth conditions. This is a fundamental characteristic employed widely in the natural environment in order for bacteria to survive harsh environmental conditions over a long period to survive. In spite of the importance of the VBNC state in microbiology, we know little about the molecular makeup. In this study, using the model organism Vibrio parahaemolyticus, we resolved that distinct subpopulations of bacteria exist in the VBNC state that have a different resuscitation potential and have a distinct proteomic profile. We have also discovered that deletion of the gene encoding the enzyme lactate dehydrogenase (lldD) causes the cells to enter the VBNC state significantly faster than the wild-type, and adding lactate to VBNC cells extended their resuscitation potential window. The ability for bacteria to survive in the VBNC state might be linked to their ability to overcome oxidative stress.”.**

3. Line 53-65: the first paragraph of the intro needs reference for each statement.

**We have now added in several references to support statements made in the first paragraph of the introduction.**

4. L230: different number of "event" were sorted from P1 and P2, could that affect the results of the infection assay?

**Generally, for FACS, collecting more cells during collection experiments is better for statistical power. We found it could take 1-2hrs to collect 50,000 cells/events of the P2 population off the FACS machine, as they were a rarer subpopulation. By contrast, it took less than 2 minutes to collect 250,000 cells/events from P1 population off the FACS. As a result, we collected different numbers of events for the populations and normalised the P1 population counts as a percentage of those that resuscitated from the total number collected. To reflect this we have now changed Line 233 from “To test resuscitation capabilities of individual VBNC subpopulations we used FACS and collected 50,000 or 250,000 cells/events from the P1 and P2 populations at time point T12 and T50.” to read “To test resuscitation capabilities of individual VBNC subpopulations we used FACS to collect 250,000 or 50,000 cells/events from the P1 and P2 populations respectively at time point T12 and T50. We have presented the number of resuscitated cells as a percentage of the total number collected.”.**

**For the infection assay, once we had determined how many cells in the population could be resuscitated we normalised the inoculum to account for this. On line 270, we state “When approximately 10^4^ CFU of VBNC subpopulation P1 were resuscitated and the cells were injected into larvae, we found they did not kill larvae within 48 h. Conversely, when approximately 10^4^ CFU of VBNC subpopulation P2 were resuscitated and cells were injected into larvae, all of the larvae died within 24 h.” Here, we collected more P1 cells, determined how many had been resuscitated and then normalised this number so that it would be 10^4^ CFU of resuscitated cells.**

L242 and 249: I guess the author means Fig 4A, not Fig 4?

**Yes, this is correct we did mean Fig4A on both these occasions. However, whilst addressing**

**other points above, we have now changed this to Fig 4B.**

L 259-263: this was hard to understand, the text should be revised. I don't see the link with the other part of this paragraph.

**We have now changed the line 262-264 from ‘This indicates that subpopulations of VBNC cells alone are not able to cause disease in *G. mellonella.’* to now read ’Even though death was seen when the microcosm was injected as a whole, larvae death was not observed when subpopulations of VBNC cells were separated and then injected into larvae.’**

L337: what does "more protein made redundant" means?

**This means that proteins were present in the samples but were not required for the function in this case the VBNC state**. Overall, we observed an increasing number of proteins were downregulated in the VBNC state as it progressed from T12 to T50, suggesting they do not play a role in the formation of VBNC cells.

Fig 6C is unclear. Are does proteins shared between P1 and P2?

**Yes, these core proteins means that they are shared between P1 and P2 and during the different time points tested. We have now changed the legend describing Figure 6C from “C: shows core proteins at T12 and T50 in subpopulations P1 and P2.” To read “ C: shows core proteins that are shared in subpopulations P1 and P2 at both time points T12 and T50”.**

Fig 8B: the authors always show the curve until the population becomes all VBNC. Why not do the same for this?

**At the time of carrying out these experiments, it was not possible to take these to completion after March this year because of the enforced shutdown of the University labs in the UK due to SARS-CoV-2. We have now carried these experiments out again to completion and have replaced Fig 8B with an updated graph. We have also changed the text on line 397-399 that read “Addition of sodium pyruvate to the microcosms was able to restore the rate of entry into the VBNC state of the RIMD2210633:*ΔlldD* for the first 17 days of the experiment (Fig 8B).” to read “ Addition of sodium pyruvate to the microcosms was able to restore the rate of entry into the VBNC state of the RIMD2210633:*ΔlldD* to that of the wild type (Fig 8B).”.**

L424-425: "For the present studies we have consistently used fresh cultured bacteria." is in contradiction with the methods for the microcosm that mentioned 5 day old culture and 14 days stored culture.

**We have changed this line (now Line 450) from “For the present studies we have consistently used fresh cultured bacteria.” To read “For the present studies we have consistently used bacteria that had been cultured <5 days from freezer stocks.” For continuity we have also changed line 446 from “In this study, when fresh cultures were used to prepare microcosms…” to now read “In this study, when fresh cultures (<5 days from culturing from freezer stocks) were used to prepare microcosms…”.**

L468: Fig 4B?

**This has been corrected (see above).**

Reviewer #2:

1. In the intro, the transition between using transcriptomic and proteomic is not well supported. If transcriptomics cannot identify unannotated genes that are important for VBNC, it is unlikely that proteomics can.

**We have changed the line 97-99 from “Collectively, the transcriptome studies on VBNC state in Vibrio species [16, 17] are useful but, crucially, do not identify unassigned genes which may play a role in VBNC formation.” to now read “Collectively, the transcriptome studies on VBNC state in Vibrio species [27, 28] are useful but, crucially, they do not identify genes where the function is unknown that may play a role in VBNC formation.”.**

2. The low image quality of multiple figures significantly impacts the readability of the manuscript. Some figures were unreadable.

**Figures 2, 4, 5, 6 and 7 have been amended to higher resolution and include suggestions from all reviewers to make text larger and clearer.**

3. The authors need to provide experimental details as what were considered as “stained as live and metabolically active” in the main text.

**Boiled and log phase cultures were used to set the gates and this information has now been included in supplementary S9 Figure and referenced to in Lines 604-606.**

4. Can the author provide possible explanation why P2 has high PI signal but intact membrane?

**The subpopulation P2 does not have a higher PI signal; it has a high Syto9 signal. We apologise for this confusion and have changed Figure 2 so that the X axis now reads Y610/20A (Syto9). We realise also that this confusion may have arisen by our erroneous description in the methods section at lines 604-606 that read “Particle fluorescence was measured at 488 nm excitation, 530 ± 30 nm emission for the RSG and Syto9 stain, and 561 nm excitation, 610 ± 20 nm emission for the PI stain”. We have now corrected this to read “Particle fluorescence was measured at 488 nm excitation, 530 ± 30 nm emission for the PI stain, 610 ± 20 nm emission for the Syto9 stain.”.**

5. How did the author determine the bacteria in the seafood were V. parahaemolyticus?

**Using FACS we collected the cells that were in the P2 gated population and plated them onto selective TCBS plates. All colonies appeared as green, sucrose negative, 2-3mm in size typical of *V. parahaemolyticus*. We tested >20 colonies for the species marker toxR by PCR and all presumptive *V. parahaemolyticus* were positive. We have added in lines 293-396to now read “Cells from P1 and P2 gated areas were collected using FACS and non-resuscitated cells could not be grown on agar while those treated with the resuscitation step were culturable and were identified as *V. parahaemolyticus* (Fig 5F) using ToxR species targeted PCR.” to address the reviewers comment.**

6. What percentage of V. parahaemolyticus population in the seafood samples were VBNC? If all detected V. parahaemolyticus were VBNC cells, and these cells cannot be resuscitated in the host, does entering VBNC state mean a pathogenesis dead-end for V. parahaemolyticus? The authors should expand a bit on this in the discussion.

**It is not possible to work out what percentage of *V. parahaemolyticus* in the seafood sample was VBNC as we did not detect any that were culturable without resuscitation. We can only say there were <20CFU/25g using our limit of detection. If VBNC cells cannot be resuscitated inside the host under these conditions as we show in this study, then a pathogenesis dead end is a distinct possibility. However, the risk to the consumer would be if there were a lag period or a change in conditions, which allowed the VBNC cells in the food product time to resuscitate.**

**We have added text to lines 494-499 to reflect this comment. “If VBNC cells cannot be resuscitated inside the host under these conditions as shown in this study, then the pathogenic nature of the *V. parahaemolyticus* would appear to be curtailed. However, the risk to the consumer would be if there was a lag period or a change in conditions, which then allowed the VBNC cells in the food product time to resuscitate, thus restoring pathogenesis.”**

Reviewer #3:

1. In Figure 1, I’m surprised by the low amount of variation (i.e small error bars) and apparent high level of reproducibility given the complexity of these experiments. Given the number of unknown factors that may contribute to the findings reported in Fig. 1, the authors should state explicitly how many biological replicates were performed to produce these data (this is not mentioned in the Results or Methods). If it wasn't already done, these experiments should be repeated independently at least 3 times to solidify the conclusions made in Figure 1, which sets the groundwork for the rest of the manuscript.

**There is variation in the counts of viable cells after day 0, but none that show a log-fold difference in counts. We have checked the error bars and they are correct. For some points, there are only 2 readings. The low level of variation reflects the time we spent optimising the experimental system. This included controlling many experimental steps such as avoiding damaging the cells by careless handling after centrifugation and by using loops to resuspend cells instead of pipetting up and down (which we found damaged the cells). We also carefully monitored temperatures especially during refrigeration since we noticed that fluctuations in fridge temperatures also affected the rate of VBNC formation of the microcosm. In numerous experiments we have found that most of the variation occurred at the time when cells could no longer be cultured on agar. This would vary between 30-35 days.**

**The key aspects of this experiment that set the groundwork for the rest of the manuscript were the days on which we carried out the FACS resuscitation experiment and the days on which we carried out the proteomics analysis. For this, we always used a time point that was 12/50 days after the point at which the microcosm turn VBNC (the time it took to turn VBNC did vary between replicates from somewhere between 30-35 days).**

1. In Figure 3, the images should be labelled within the figure so that the reader does not have to rely exclusively on the figure legend.

**We have added more information to Figure 3. Each panel is still labelled from A-E but with extra detail about what subpopulation that is being analysed.**

2. It would be interesting to analyse the cell morphology of the Δ*lldD* mutant

**We have carried out these experiments and have added this information into supplementary Figure 11. At T0 time point the length of Δ*lldD* (1.899±0.56 µm) was larger in length size compared to T0 timepoint of the wildtype RIMD2210633 (1.26±0.1 µm). When the** **Δ*lldD* mutant was put into the VBNC state, it showed at T12 that the morphology of VBNC cells had changed. Overall there were fewer large coccoid cells in the P2 population of the Δ*lldD* mutant (2%) compared to the P2 population of RIMD2210633 (10%). The large coccoid cells present in the P2 population of the Δ*lldD* mutant were smaller (length 3.16±0.15 µm width 1.76±0.2 µm) than RIMD2201633 large coccoid cells (length 6.34 ± 1.10 µm, width 4.30 ± 0.48 µm). Also, in the P2 population of the Δ*lldD* mutant, there were larger numbers of filaments or chains of cells present.**

**We have now included this data to the supplementary section as S11 Figure and we have inserted a section of text into the results to reflect this data at lines 394-395 and 400-407.**

3. In Figure 8C, the authors should repeat this experiment (addition of sodium lactate) using the ΔlldD mutant.

**We have now carried these experiments out and have answered this comment below in comment number 4.**

4. Does inactivation of TCA cycle metabolism gene(s) also lead to cells entering VBNC state significantly earlier (like ΔlldD mutant)? Can you resuscitate VBNC stage of cells with other metabolites such as glycerol?

**We thank the reviewer for this comment. We did test the wild type RIMD2210633 microcosm with other substrates including sodium acetate, sodium pyruvate and sodium lactate. We found that only sodium lactate was able to resuscitate the VBNC cells. We agree with the reviewer that it would be interesting to test the proposed assays on the lldD mutant. We have now tested the lldD mutant with sodium pyruvate, sodium acetate and sodium lactate to see if these compounds are able to resuscitate VBNC cells. We found that none of these compounds including sodium lactate was able to resuscitate the lldD mutant when they were in the VBNC state. To reflect these experiments that were carried out we have changed lines 421 to 432 from “We investigated whether the addition of sodium lactate would enable the resuscitation of mid-to late stage VBNC cells, when thermal shift methods alone were not sufficient. The addition of 2 mM of sodium lactate resuscitated mid VBNC stage cells (36 days after cells had become unculturable in the microcosm) enabling some cells to be cultured, whilst control VBNC cells incubated in PBS were not resuscitated (Fig 8C).”to now read “We investigated whether the addition of sodium lactate, sodium pyruvate and sodium acetate would enable the resuscitation of mid-to late stage VBNC cells, when thermal shift methods alone were not sufficient. The addition of 2 mM of sodium lactate resuscitated mid VBNC stage cells (36 days after cells had become unculturable in the microcosm) enabling some cells to be cultured, whilst control VBNC cells incubated in PBS were not resuscitated (Fig 8C). The addition of other oxidative compounds including sodium pyruvate and sodium acetate did not resuscitate RIMD2210633 VBNC cells.**

**When we investigated whether the addition of sodium lactate, sodium pyruvate and sodium acetate would resuscitate RIMD2210633:*ΔlldD* VBNC cells in mid-late stage we found that none of these compounds were able to turn the VBNC cells to culturable forms as seen with the wildtype.”**

5. Does a ΔlldD mutant have altered virulence in the larval infection model? The authors should address this question experimentally.

**We have injected the RIMD2210633:*ΔlldD* into larvae at a dose of 10^5^ CFU per larvae and we saw no significant difference in killing after 48 hours compared to the wild type RIMD2210633. We have now added this data to the supplementary data (S10 Figure).**
